# The lockdown effects on a pediatric obese population in the COVID-19 era

**DOI:** 10.1186/s13052-021-01142-0

**Published:** 2021-10-18

**Authors:** M. Valenzise, F. D’Amico, U. Cucinotta, C. Lugarà, G. Zirilli, A. Zema, M. Wasniewska, G. B. Pajno

**Affiliations:** Department of Human Pathology of Adulthood and Childhood, University of Messina, Messina, Italy

## Abstract

**Background:**

The social consequences of COVID-19 pandemic are universally known. In particular, the pediatric population is dealing with a radical lifestyle change. For some risk categories, such as overweight or obese children, the impact of home confinement has been greater than for others. The increased sedentary life, the wrong diet and social distancing have stopped the chance of losing weight.

The aims of this study were to analyse the impact of COVID-19 lockdown on the behavior changes in a obese pediatric population and to explore the correlation between the new lifestyle and the level of parental instruction.

**Methods:**

Data show features of 40 obese and overweight pediatric patients of our Clinic in Messina (Italy). We evaluated weight, height, BMI and other biochemical parameters: total cholesterol, HDL, LDL, triglyceride, transaminases, glycemia and insulinemia. After the lockdown, we contacted all patients in order to get some information about diet, physical activity and sedentary lifestyle changes in correlation to the level of their parents’ instruction. Additionally, we also evaluated 20 children twice from a clinical and laboratory perspective.

**Results:**

The study showed an increase of daily meals during COVID-19 lockdown (3.2 ± 0.4 vs 5 ± 1, *P* < 0.001). In particular, children whose parents have primary school diploma ate a greater significant number of meals during the lockdown, compared to those who have parents with secondary school diploma (*P* = 0.0019). In addition, the 95% of patients did low physical activity during the lockdown and the 97.5% spent more time in sedentary activity. Even if BMI’s values don’t show significant differences, they have increased after the lockdown. We didn’t find any correlation between biochemical parameters before and after the lockdown.

**Conclusion:**

The lockdown has had bad consequences on good style of life’s maintenance in overweight and obese children. The absence of a significant correlation between the worsening of biochemical parameters and the lockdown doesn’t allow to exclude any long-term consequences. It’s safe to assume that, if the hours spent in sedentary activity and the number of meals don’t diminish, there will probably repercussion on the biochemical parameters.

## Background

COVID-19 lockdown period has had an important impact on the world population. This current situation is causing several consequences not only linked to health, but also to the need of lifestyle changes. On 11th March 2020, the World Health Organization declared the status of global pandemic [1] and many nations adopted measures in order to contain future infections. For example, in Italy the first lockdown started from 11th March 2020 [2] and lasted until the end of May: during this period numerous shops closed and group activities were forbidden. It had a great impact on normal habits but the biggest price has been paid from the pediatric population, who has seen a radical change in their lives. Social distancing, the impossibility to stay physically at school or to spend time with other people gave a huge incentive to social inequality and to health problems, including mental and eating disorders [3, 4]. In particular, for some risk categories, such as overweight or obese children, the consequences were greater. Not only the increased sedentary life, the wrong diet and the social distancing have prevented the chance of losing weight, but they also worsened the general life conditions. The opportunities to do physical activity dropped dramatically due to the COVID-19 pandemic, especially for children who lived in small apartments of urban areas [5]. In addition, recent studies suggest that the consumption of chips, red meat, and sugary drink increased significantly during the lock-down [6]. Conversely, the healthy adult population examined showed an improvement in the quality of the diet in the early phase of the COVID-19 lockdown making us understand that the home confinement was harder for the pediatric population [7].

However, it’s also true that the fight against overweight and obesity in the pediatric population is extremely hard but essential for the health condition of future generations and, therefore, the problem can’t be overlooked.

A lot of studies show us how high levels of total cholesterol, LDL, triglycerides, glycemia and low level of HDL in the overweight and obese children are considered as important cardiometabolic risk factors, especially for boys and young men [8]. We must not neglect either the levels of transaminases, like GOT and GPT, for the well-known association between high level of them and the NAFDL, gateway to liver fibrosis [9].

Moreover it’s commonly known that an inadequate intake of some nutrients like Vitamins A, D and E, minerals zinc, selenium and essential fatty acid, as observed in pediatric obese population, could cause a decrease of resistance to infections [10], while an equilibrated dietary intake can also protect against an excessive inflammatory response to SARS-Cov-2 infection (frequent in the obese [11]) and, consequently, it can guarantee a better outcome [12, 13].

Due to these reasons, the lifestyle changes constitute a real social alarm. The aim of this study is to analyse the effects of the lockdown on the behaviour changes of the obese pediatric population and the correlation between the new lifestyle and the level of parents’ instruction.

## Methods

### Study design and rationale

The population taken into consideration consists of a group of obese and overweight pediatric patients analysed before the COVID-19 pandemic, followed in our Clinic in Messina, Italy. Data were obtained retrospectively. The inclusion criteria were: chronological age between 2 and 18 years, BMI SD > 1; the exclusion criteria were genetic and endocrinological cause of obesity, diagnosis of chronic diseases, chronic therapies and smoking. Through a telephone interview conducted by two doctors and a dietitian after the lockdown, we could evaluate diet, physical activity, sedentary lifestyle changes during the period at home and the level of their parents’ instruction. We asked how many hours children spent doing various activities such as playing videogames, attending online lessons, watching television, additionally to the number of meals before and after the lockdown per day. We also divided the population into 3 groups: the first one included patients whose parents had a primary school diploma; the second one contained patients whose parents had middle school diploma; the third one was made up of patients whose parents had high school diploma or a degree. In addition, we evaluated some of them (20 children) twice from a clinical and laboratory perspective.

According to WHO classification, we considered a child to be overweight if BMI was between 85th and 94th percentiles (from 1 to 1,99 DS), obese if BMI was between 95th and 98th percentiles (from 2 to 2,99 DS) and severely obese when BMI was higher than 99th percentiles (> 3 DS) [14], using Cacciari’s Growth Charts [15, 16].

The parameters evaluated were weight, height, BMI and other biochemical parameters: total cholesterol, HDL, LDL, triglyceride, transaminases, glycemia and insulinemia. Referring to the lipids’ levels, we considered normal values < 170 mg/dl for total cholesterol, > 45 mg/dl for HDL, < 110 mg/dl for LDL, < 75 mg/dl (0-9 years old) and < 90 mg/dl (10-19 years old) for triglyceride. We considered borderline values 170-199 mg/dl for total cholesterol, 40-45 mg/dl for HDL, 110-129 mg/dl for LDL, 75-99 mg/dl (0-9 years old) and 90-129 mg/dl (10-19 years old) for triglyceride. Lastly, we considered pathological values > 200 mg/dl for total cholesterol, > 45 mg/dl for HDL, > 130 mg/dl for LDL, > 100 mg/dl (0-9 years old) and > 130 mg/dl (10-19 years old) for triglyceride [17].

For transaminases values, detected by spectrophotometry, we considered pathological level of GOT > 27 U/L for female and > 32 U/L for male; we considered pathological level of GPT > 26 U/L for female; > 35 U/L for male. It’s proven that these values are associated with a high risk of NAFLD [9, 18].

Regarding fasting glycemia, we described a situation of fasting glucose intolerance when we had a glycemia of 100-125 mg/dl. We considered diabetes a glycemia > 126 mg/dl [16].

At the end, for fasting insulinemia, we considered pathological values > 16.7 μIU/ml [19].

### Statistical methods

Demographic characteristics, like age, sex, weight, height, BMI and laboratory data like total cholesterol, HDL, LDL, triglyceride, transaminases, glycemia and insulinemia has been done before and after the lockdown as regards a descriptive and comparative analysis. Another descriptive and comparative analysis has been conducted between the grade of parents’ instruction (primary school diploma, middle school diploma and high school diploma) and the number of meals per day before and after the lockdown.

Descriptive statistics of the patients’ characteristics are provided as mean and SD for continuous variables and frequency and percentages for categorical variables (IC = 95%).

We used Kolmogorov-Smirnov’s test to study variables distribution, with a non-parametric approach. For the categorical variables, it has been used Pearson’s Test, whereas the U Mann-Whitney’s Test for continuous variables. A statistical significance was found when the *p*-value was < 0,05. All statistical analysis were performed using software Statistical Package for the Social Science (SPSS) 23.0 for Windows (IBM Corp. SPSS Statistics).

## Results

The examination involved 40 patients, 17 females (42.5%) and 23 males (57.5%). Before the lockdown, the group had a mean age of 11.6 ± 3.3, a mean weight of 68 ± 19.8 kg and a mean height of 143 ± 29 cm.

The analysis of daily meals before the lockdown showed a mean number of 3.2 ± 0.4, statistically lower than the value of daily meals during the lockdown (5 ± 1.3, *p*-value < 0,001).

We also observed that 38 patients (95%) spent less time doing physical activity during the lockdown and 39 patients (97,5%) admitted a longer time spent in sedentary activity than before, such as watching television and playing videogames.

In a second phase, we classified these patients according to the level of their parents’ instruction in 3 groups (primary school diploma, middle school diploma, high school diploma or degree). The descriptive analysis didn’t show any statistically significant difference as regards the number of daily meals between the three groups before the lockdown. The mean values were 3.2 ± 0.4 vs 3.03 ± 0.5 vs 3.1 ± 0.3. On the contrary, we noticed an increased mean number of meals after the lockdown in the 3 categories that were respectively 6 ± 0.7 vs 5.3 ± 1.2 vs 4.4 ± 1.3 respectively. In particular, the group of parents with primary school diploma had a significantly higher mean number of meals than those with high school diploma/degree (6 ± 0.7 vs 4.4 ± 1.3, *p*-value = 0.019). No significant difference of meals number was found between the group of parents with elementary school diploma and the group of parents with middle school diploma (6 ± 0.7 vs 5.3 ± 1.2, *p*-value = 0.331) (Table 1).

**Table 1:** Daily meals before and after lockdown according to the grade of parents’ instruction

	Elementary school (Group 1)	Middle school (Group 2)	*P*-value Group 1 vs 2	Elementary school (Group 1)	High school and more (Group 3)	*P*-value Group 1 vs 3
Before lockdown	3.2 (±0.4)	3.3 (±0.5)	0.836	3.2 (±0.4)	3.1 (±0.3)	0.672
During lockdown	6 (±0.7)	5.3 (±1.2)	0.331	6 (±0.7)	4.4 (±1.3)	0.019

Before the lockdown, the whole population analysed had a mean BMI level of 30.9 ± 5.4, mean GOT and GPT levels respectively of 20.5 ± 27.3 U/L and 28.4 ± 25.3 U/L, a mean glycemia level of 85,7 ± 12 mg/dl, a mean insulinemia level of 25,1 ± 14,5 μIU/ml, a mean total cholesterol level of 181.4 ± 32 mg/dl, mean HDL and LDL levels respectively of 48.4 ± 12.8 mg/dl and 107.5 ± 28.6 mg/dl and a mean triglyceride level of 108.6 ± 33.9 mg/dl.

After the lockdown, 20 patients were evaluated again with a mean age of 12.4 ± 2.6. For this group, the mean value of BMI before the lockdown was 30.2 ± 4, which become 32 ± 5.5 after the lockdown. Despite the increase of this parameter, no significant difference was found (*p*-value = 0.0339). The mean values of glycemia and insulinemia before the lockdown were respectively 83.5 ± 13.4 and 25.2 ± 16 μIU/dl. The same parameters after the lockdown were 83.9 ± 10.9 mg/dl and 21.4 ± 9.3 μIU/ml. The mean GOT and GPT levels were 24.2 ± 13.9 U/L and 28.4 ± 28.7 U/L before the lockdown and 20.9 ± 8 U/L e 24 ± 13.9 U/L after the lockdown. The mean levels of total cholesterol were 183 ± 31.3 mg/dl before the lockdown and 167.8 ± 22.6 mg/dl after the lockdown. The mean levels of HDL and LDL were 50.4 ± 11.6 mg/dl and 106.4 ± 27.5 mg/dl before the lockdown and 48.3 ± 9.3 mg/dl and 99.8 ± 18.5 mg/dl after the lockdown. The mean levels of triglyceride were 104.4 ± 33.7 mg/dl before the lockdown and 94.9 ± 26.5 mg/dl after. No significant difference was found between these laboratory parameters before and after the lockdown (*p* > 0.05) (Table 2).

**Table 2:** Laboratory parameters before and after the lockdown

	Before lockdown	After lockdown	*P*-value
Age	11.5 (±2.7)	12.4 (±2.6)	0.219
BMI (kg/m^2^)	30.2 (±4.0)	32.0 (±5.5)	0.339
Insulinemia (μIU/dl)	25.2 (±16.0)	21.4 (±9.3)	0.782
Glycemia (mg/dl)	83.5 (±13.4)	83.9 (±10.9)	0.539
GPT (U/L)	28.4 (±28.7)	24.0 (±13.9)	0.954
GOT (U/L)	24.2 (±13.9)	20.9 (±8.0)	0.686
Total Cholesterol (mg/dl)	183.0 (±31.3)	167.8 (±22.6)	0.089
HDL Cholesterol (mg/dl)	50.4 (±11.6)	48.3 (±9.3)	0.525
LDL Cholesterol (mg/dl)	106.4 (±27.5)	99.8 (±18.5)	0.838
Triglyceride (mg/dl)	104.4 (±33.7)	94.9 (±26.5)	0.351

## Discussion

The necessity to change our lifestyle due to the COVID-19 lockdown forced us to reduce hours of physical activity and to change our diet [20, 21]. Alarmed by some studies that reported a deterioration in the quality of life [22], we focused our attention on the obesity question to understand how the lockdown influenced the possibility to lose weight and the improvement of quality of life in our territory. We all know that obesity is such an important problem that the WHO decided to coin the term “globesity”. Childhood obesity leads to an adulthood mostly characterized by diabetes and cardiovascular problems with high costs for public health and deterioration of the life quality. In addition, a family history of obesity, DM2, high blood pressure and dyslipidaemia is associated with earlier and more severe forms of obesity [23]. In few words, the childhood obesity question involves the individual and the whole society, with a great impact in the reorganization of welfare services.

In particular, the global OECD data showed for Italy a 4th position for prevalence of overweight and obese children [24] despite being a country of the Mediterranean area with an excellent balanced diet. It may be caused by globalization which has also brought the junk food culture. Unfortunately, the current pandemic didn’t provide the possibility to change this situation. As we observed in our study, the number of daily meals during the lockdown has significantly increased (*p* < 0.001). It could be explained by the consumption of caloric and high-energy foods probably eaten because of boredom or stress caused by the limitations lockdown-related.

Analysing the number of daily meals according to the grade of parents’ instruction, our data is similar to other studies [25]. The number of daily meals during the lockdown is significantly higher in the group of parents with elementary school diploma than the one with high school diploma. No significant difference was found between the other groups. This difference could be explained considering the deeper awareness of a correct diet and the consequences of overweight in people with a higher level of instruction.

In addition, almost the totality of the examined population reported an important reduction of the hours spent doing physical activity and admitted to spend more time in sedentary activity, as we can also observe in other studies [6].

The level of BMI after the lockdown didn’t have a significant correlation with the other value before the lockdown. However, the little increase after the period at home must alarm us.

We didn’t find a significant correlation between the biochemical parameters before and after the lockdown. However, long-term consequences can’t be excluded. If the habits change and the higher consumption of junk food persists, in the next months we will be able to witness repercussion also on a biochemical point of view.

Our study has some limitations, like the small sample analysed. For a deeper study, it would be necessary to examine trend of a greater sample, considering that there was a second lockdown for lots of countries in the winter of 2021, including Italy. This second period of home confinement might have worsened the consequences on the pediatric population from a healthy and psychological point of view. Despite this fact, our study shows that the condition of obese children deserves a deeper attention due to the lockdown.

## Conclusion

The study described allows us to say that COVID-19 worsens also the situation of overweight and obese pediatric population. The prohibition of leaving the house had not only stopped the possibility of losing weight but it also created the condition for a worse adult life. During the lockdown, stress due to the pandemic brought a lot of people to find consolation in junk food, including obese and overweight children. The higher consumption of caloric food with a reduction of physical activity shows also in this study a particular attention on a problem of great importance for the future.

It’s a common belief that doing something now so as to contrast the excess of weight will be helpful in terms of reducing the complications that an obese child might have in the future [26, 27]. In this context, the COVID-19 pandemic has only exacerbated all the risk factors [5].

We can also deduce that the level of parents’ instruction plays a crucial role. The higher number of daily meals eaten by patients whose parents have elementary school diploma suggest the necessity to improve the awareness of this health problem. An important solution could be an improvement of social sensibility with awareness campaigns, teaching at school, increasing attention from pediatricians and promoting physical activity [28, 29].

Acting quickly and effectively, trying to change the mentality of the general population and adopting a prevention policy which involves doctors, schools and families represents the key to solve this problem.
